# TMS Over V5 Disrupts Motion Prediction

**DOI:** 10.1093/cercor/bht297

**Published:** 2013-10-23

**Authors:** Petra Vetter, Marie-Helene Grosbras, Lars Muckli

**Affiliations:** 1 Centre for Cognitive Neuroimaging, Institute of Neuroscience and Psychology, College of Medical, Veterinary and Life Sciences, University of Glasgow, Glasgow G12 8QB, UK; 2 Current address: Department of Neuroscience, Laboratory for Behavioral Neurology and Imaging of Cognition, Medical School and Swiss Center for Affective Sciences, University of Geneva, Geneva 1205, Switzerland

**Keywords:** apparent motion, double-pulse TMS, hMT+, predictive coding, V1

## Abstract

Given the vast amount of sensory information the brain has to deal with, predicting some of this information based on the current context is a resource-efficient strategy. The framework of predictive coding states that higher-level brain areas generate a predictive model to be communicated via feedback connections to early sensory areas. Here, we directly tested the necessity of a higher-level visual area, V5, in this predictive processing in the context of an apparent motion paradigm. We flashed targets on the apparent motion trace in-time or out-of-time with the predicted illusory motion token. As in previous studies, we found that predictable in-time targets were better detected than unpredictable out-of-time targets. However, when we applied functional magnetic resonance imaging-guided, double-pulse transcranial magnetic stimulation (TMS) over left V5 at 13–53 ms before target onset, the detection advantage of in-time targets was eliminated; this was not the case when TMS was applied over the vertex. Our results are causal evidence that V5 is necessary for a prediction effect, which has been shown to modulate V1 activity (Alink et al. 2010). Thus, our findings suggest that information processing between V5 and V1 is crucial for visual motion prediction, providing experimental support for the predictive coding framework.

## Introduction

The brain is constantly confronted with a wealth of sensory information that needs to be processed fast and efficiently to guide our actions. One way of reducing this massive processing effort is to predict incoming sensory information based on previous experience, so that expected information is processed efficiently and enough resources can be allocated to novel information. The framework of predictive coding captures this idea both on a theoretical level (Mumford 1992; Friston 2010) and a computational level (Rao and Ballard 1999). Predictive coding states that the brain constantly models the world based on context and memory, and predicts the sensory input. Such predictive models are created in higher cortical areas and communicated through feedback connections to lower sensory areas. Feed-forward connections communicate an error signal, that is, the mismatch between the predicted information and the actual sensory input (Rao and Ballard 1999). The predictive model is then constantly updated according to this error signal. Despite being theoretically well founded, direct experimental evidence for predictive coding is still sparse.

We developed an experimental paradigm to study predictive coding in the visual system based on the well-known illusion of apparent motion in which flashing 2 stationary stimuli in rapid succession creates the illusion of a single moving token. The creation of the illusory motion token can be regarded as the instantiation of a prediction: given the context of 2 flashing stimuli, the brain infers the presence of a single moving token. Using brain imaging, we demonstrated that V1 neurons retinotopically responsive to the apparent motion trace are activated during apparent motion as if real motion was present, despite the absence of actual feed-forward stimulation (Muckli et al. 2005). This effect may be explained by visual motion area V5/human motion complex, human medial temporal complex (hMT) communicating the prediction of a moving token to V1 via feedback connections (Goebel et al. 1998; Muckli et al. 2002; Silvanto et al. 2005; Larsen et al. 2006; Sterzer et al. 2006; Ahmed et al. 2008; Wibral et al. 2009; Frégnac et al. 2010). Furthermore, we showed that the creation of a predictive signal on the apparent motion trace is spatio-temporally specific: Targets flashed on the apparent motion trace in-time with the illusory motion token are detected better than that flashed out-of-time (Schwiedrzik et al. 2007; Vetter et al. 2012). In addition, this effect modulates V1 where in-time targets elicit lower activity levels than out-of-time targets (Alink et al. 2010). Both findings corroborate the idea of predictive coding: Spatio-temporally predictable targets are more efficiently processed than unpredictable targets and elicit a lower signal in V1 due to their smaller error signal.

In this experiment, we sought to establish causal evidence for the role of V5 in creating predictive motion signals. We used the paradigm by Schwiedrzik et al. (2007), in which we flashed spatio-temporally predictable or unpredictable targets on the apparent motion trace and disrupted V5 with transcranial magnetic stimulation (TMS) before, during, or after target presentation. We hypothesized that if V5 projections to V1 are necessary to create a spatio-temporal prediction, then TMS over V5 should interfere with the detection advantage of predictable targets.

## Materials and Methods

### Subjects

Seventeen subjects (6 females, mean age 24.5) were included in the study for TMS over V5. As a control group we tested another 17 subjects (7 females, mean age 26.6), who received TMS over the vertex. All were right-handed, had a normal or corrected-to-normal vision, and no history of neurological or psychiatric disorder. Subjects completed a screening questionnaire for contraindication to TMS, signed informed consent, and were paid for their participation. The study was approved by the ethics committee of the College of Science and Engineering, University of Glasgow.

Another group of 15 subjects (11 females, mean age 24.4) was recruited for a purely behavioral control experiment (see below). These subjects also signed informed consent.

### TMS Experiment

#### Visual Stimuli

**Figure 1.**
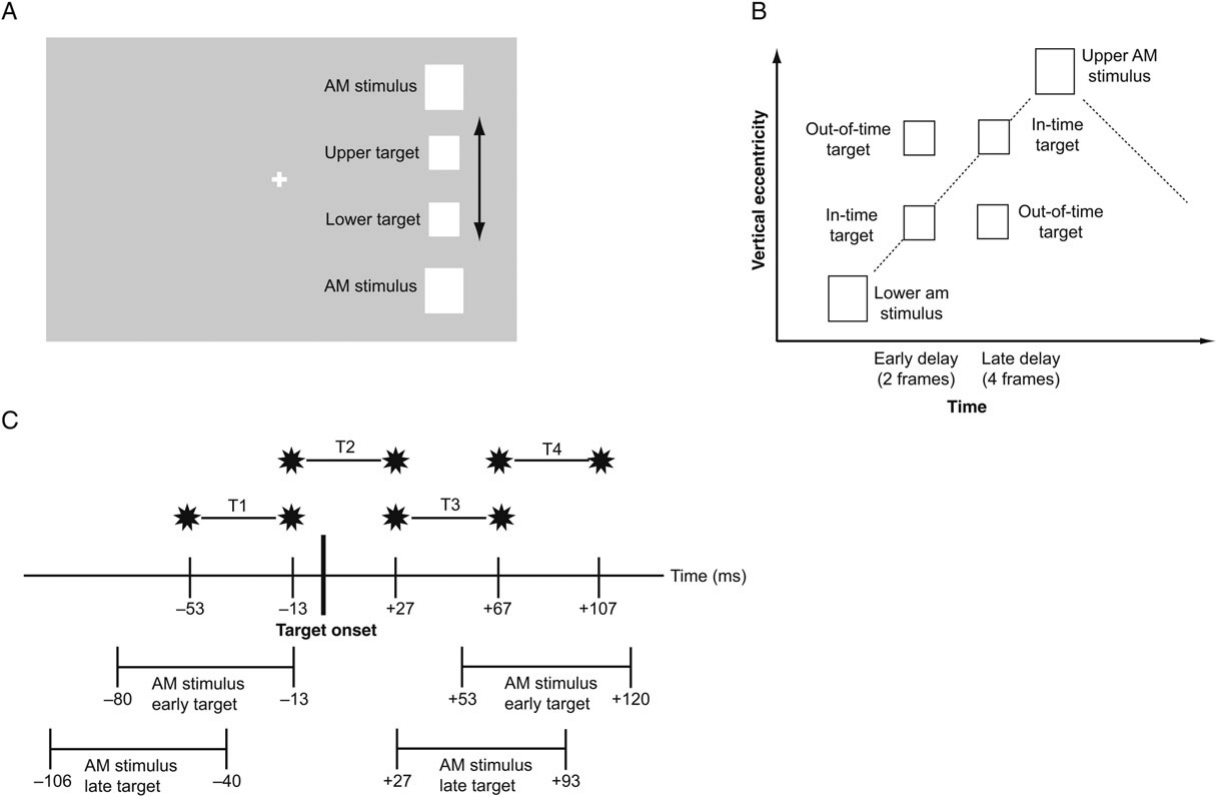
Stimuli and experimental design. (*A*) Schematic depiction of the visual stimulus (not in scale). The apparent motion inducing stimuli flashed alternately at a frequency of 3.75 Hz in the periphery. We flashed targets on the apparent motion trace at either an upper or a lower position. (*B*) Space-time plot of on apparent motion cycle. Targets were flashed either in-time with the illusory motion token, that is, at the expected place and time assuming a linear motion trajectory, or out-of-time with the token, that is, spatio-temporally incongruently. (*C*) Time windows of TMS. We applied double-pulse TMS either before target onset (T1), around target onset (T2), shortly after target onset (T3), or slightly later (T4). The interpulse interval was 40 ms.

Two apparent motion stimuli (white squares, 2.5° each, 14.8° vertical distance) were flashed in rapid succession (67 ms, interstimulus interval [ISI]: 67 ms, 3.75 Hz) at 8.5° to the right of the central fixation cross (0.06°, see Fig. 1*A*). The target (white square, 2°) was flashed for one frame of 13.3 ms at either an upper or lower position on the apparent motion trace (2.3° distance from the midline). The target was presented either spatio-temporally congruent with a linearly moving illusory token (in-time targets) or incongruent (out-of-time targets), that is, at the same time but at the wrong position (see Fig. 1*B* and Supplementary Video clips). Each cycle of apparent motion lasted 20 frames (267 ms): 5 frames of upper apparent motion stimulus, 5 frames ISI, 5 frames of lower apparent motion stimulus, and 5 frames ISI. The targets were displayed in either the second or the fourth frame of the ISI. Each trial consisted of 10 cycles of apparent motion, with the target flashed in cycles 4–6 (randomized). The target appeared equally often in either upward or downward apparent motion. Target timing and position were counter-balanced across trials. There was no intertrial interval and apparent motion stimulation continued for blocks of 16 trials without interruption. Thus, start and end of the individual trials were not perceptible for subjects. Only in TMS trials, the TMS pulse indicated the presence of a trial. After blocks of 16 trials (42.7 s), apparent motion was interrupted for 25 s with a natural scene display to prevent apparent motion breakdown due to adaptation and to give subjects a rest from TMS. The optimal target contrast was assessed for each individual by varying the gray level of the background in a brief behavioral pretest. Stimuli were presented on a 16-inch Dell Trinitron CRT monitor (75 Hz, resolution 1024 × 768).

#### Task and Procedure

The task was to keep central fixation and to detect targets on the apparent motion trace, indicating their presence by a key press. Central fixation was monitored with eye-tracking (EyeLink) throughout the experiment. Before the TMS session, a pretest session was run to determine the optimal target contrast for each participant, to familiarize subjects with the task, and to determine the main prediction effect (a higher detection rate for in-time targets). Subjects who did not show a detection difference between in-time and out-of-time targets in the pretest were excluded from the experiment (*n* = 2).

#### TMS and Experimental Design

Motion area V5/hMT was mapped in the left hemisphere in each subject individually in a separate functional magnetic resonance imaging (fMRI) session prior to the TMS experiment (see below). Despite stimulation at different time windows provides a within-subject control condition, a control group was stimulated at the vertex with exactly the same parameters to ensure that our effects were not due to the audible click and the physical sensation of the TMS pulse. Vertex stimulation was chosen as it should not affect any brain tissue. Double-pulse TMS (interpulse interval of 40 ms) at 50% of the maximal stimulator output (MagStim 200 equipped with a BiStim module) was applied using a 70-mm figure-of-eight coil. A 50% stimulation level was the stimulation strength that was tolerated well by all subjects, and that did not induce phosphenes in any of our subjects. Exact coil positioning was ensured using frameless stereotaxy (BrainSight, RogueResearch) and monitored throughout the experiment. Double-pulse TMS was applied at 4 different time windows: Before target onset (T1; −53 to −13 ms), around target onset (T2; −13 to +27 ms), shortly after target onset (T3; +27 to +67 ms), and slightly later after target onset (T4; +67 to +107 ms), see Figure 1*C*. TMS timing (T1–T4) was randomized across TMS trials. TMS trials alternated with no-TMS trials to allow for sufficient time between 2 TMS pulses (range 4.1–6.3 s). Eighty percent of the trials contained a target, and of the remaining 20% without a target, half contained a TMS pulse. Subjects performed 640 trials in total (half with TMS and half without TMS), 64 for each TMS time window (half in-time targets and half out-of-time targets). The experiment was broken down into 4 runs of 15 min each to give subjects a rest and to prevent overheating of the TMS coil. The TMS session lasted for 1.5–2 h.

### Functional Localization of V5 with fMRI

#### Visual Stimuli

Localization stimuli consisted of real motion, a flickering, or a stationary stimulus presented in the right visual field at the same location as the apparent motion trace in the TMS experiment (see above). Real motion was also presented in the left visual field (same eccentricity) to isolate medial temporal (MT) area from medial superior temporal (MST) area. Stimulus properties were kept as similar to the TMS experiment as possible. The background was mid-gray. The real motion stimulus (a white square of 2.5°) moved up and down at a frequency of 2.5 Hz. The flickering stimulus consisted of a white bar (2.5° by 15°) flickering at 5 Hz. The stationary stimulus consisted of the same bar being presented for the length of the trial (16 s). During the whole run, the central fixation cross (0.06°) changed color for every 800 ms.

#### Task and Procedure

Subjects viewed the stimuli through MRI-compatible goggles (NordicNeuroLab). The task was to keep central fixation at all times, to press a button when the fixation cross turned red, and to ignore the stimuli in the periphery. Central fixation was ensured by eye-tracking. Mean task accuracy was 96.1% (standard error of the mean, SEM, 2.4). Each stimulus was presented in blocks of 16 s, 6 times pseudorandomly repeated in each run, with a 16-s fixation screen at the start and end of each run. Two functional runs were recorded, interleaved by an anatomical scan. Scanning lasted for about 40 min.

#### fMRI Data Acquisition and Analysis

Blood oxygen level-dependent (BOLD) signals were acquired in a 3-T Siemens Tim Trio (time repetition = 1.6 s, time echo = 30 ms, resolution 2 × 2 × 2 mm, 25 slices, flip angle 62°, iPAT factor 2, 12-channel coil). Data were analyzed with BrainVoyagerQX (BrainInnovation) with standard preprocessing. Each subject's left V5 was identified as peak activation of the contrast Real Motion (right) > [Flickering Bar and Stationary Bar]. By comparing Motion contralateral > Motion ipsilateral, we ensured that our ROI included MT rather than MST. The peak voxel was marked on the anatomical scan and imported into BrainSight (RogueResearch). We localized V5/hMT at mean Talairach coordinates [−43.6, −72.3, 4.9] with standard deviations [5, 6, 2.6], in correspondence to previous reports (Dumoulin et al. 2000).

### Behavioral Control Experiment

We performed an additional behavioral control experiment to assess the dependency of target detection on conscious apparent motion perception.

#### Visual Stimuli

Visual stimuli were the same as in the TMS experiment, but stimulus presentation was divided into separate trials (8 cycles of apparent motion [160 frames], with the target displayed in cycles 3–6 [randomized], resulting in a trial duration of 2.13 s length). After each trial, 2 screens prompted subjects to report whether they detected a target, and whether they perceived apparent motion. Prompts stayed on the screen until subjects responded. The fixation cross was shown for 500 ms at the beginning of each trial before apparent motion display started.

#### Task and Procedures

After each trial, subjects were prompted to make a 2-alternative forced choice first on whether they detected a target and then on whether they consciously perceived apparent motion. Trials were sorted post-hoc according to whether subjects reported apparent motion perception or not. Central fixation was monitored with eye-tracking during apparent motion display.

#### Experimental Design

Subjects performed 200 trials. In 60% of the trials, the target was displayed in the lower position (target of interest), in 10% of the trials, the target was displayed in the upper position (catch trials), and in 30% of the trials, there was no target. Half of the targets appeared in-time, half out-of-time. As in the TMS experiment, a natural scene was displayed after every 40 trials.

## Results

In the context of flashing stimuli, the brain infers the existence of a continuously moving illusory token that leads to the perception of apparent motion. This moving token can be conceptualized as a spatio-temporal prediction along the apparent motion path. Targets that appear spatio-temporally congruent with the illusory token confirm this prediction, whereas incongruent targets violate this prediction. Thus, our experimental manipulation of in-time and out-of-time targets aimed at the spatio-temporal predictability within the apparent motion context. Note that we did not manipulate expectation of the presence, position, or timing of targets across trials.

**Figure 2.**
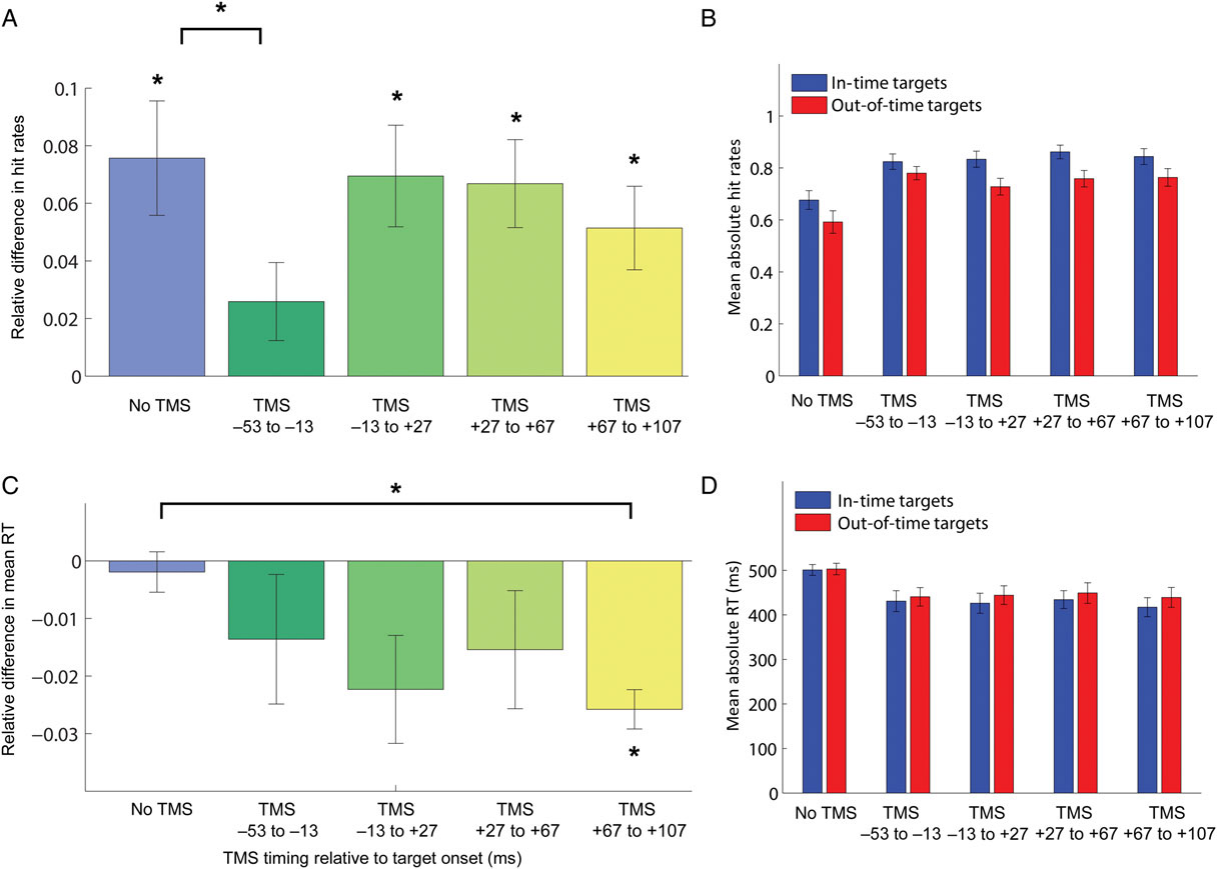
Experimental results for TMS over left V5 (*n* = 17). (*A*) Relative differences between in-time and out-of-time targets for hit rates. Positive values mean that in-time targets were better detected than out-of-time targets. Stars directly above or below the bars indicate significant (*P* < 0.003) 1-sample *t*-test results against 0 (Bonferroni-corrected). Stars above the brackets indicate *t*-test results of comparisons with the no-TMS condition (*P* < 0.05, Bonferroni-corrected). (*B*) Absolute mean hit rates (proportion of trials) for in-time and out-of-time targets. (*C*) Relative differences between in-time and out-of-time targets for reaction times. Negative values indicate that in-time targets were detected faster. (*D*) Absolute mean reaction times (ms). All error bars represent SEM.

Replicating previous results (Schwiedrzik et al. 2007; Vetter et al. 2012), predictable in-time targets were overall detected more frequently than unpredictable out-of-time targets (see Fig. 2*B* for absolute mean hit rates in all experimental conditions; repeated-measures analysis of variance [ANOVA]: *F*_1,16_ = 39.17, *P* < 0.001). In addition to previous findings, we also found that mean reaction times, across conditions, were shorter for predictable than for unpredictable targets (see Fig. 2*D* for absolute reaction times for all experimental conditions; *F*_1,16_ = 20.61; *P* < 0.001).

Overall false alarm rate was 4.2% (SEM 1.1). Given the 32 trials in each experimental condition, an average hit rate for in-time targets of 84% meant that, on average, 27 targets of 32 were detected, with an average hit rate of 76% for out-of-time targets, and 24 targets of 32 were detected. The same effects were found in the brief pretest session with a varying background contrast (hit rates: *F*_1,15_ = 11.91, *P* = 0.004; mean RT: *F*_1,15_ = 9.96, *P* = 0.007). Due to the fact that apparent motion stimulation was ongoing and start and end of the trials were not perceptible, the presence of a trial, and thus the potential presence of a target, was indicated by the TMS pulse in the TMS trials, but not in the interleaved no-TMS trials. Therefore, absolute hit rates were lower, and RTs higher in the interleaved no-TMS trials than in the TMS trials, because subjects did not expect a target as often as in the TMS trials (hit rates: *F*_4,64_ = 18.96, *P* < 0.001; RT: *F*_4,64_ = 25.22, *P* < 0.001).

However, our critical measure of the predictive effect was not absolute performance measures, but the relative performance difference between the in-time and the out-of-time target conditions because this gives an indication of the perceptual difference of predicted and unpredicted targets. Relative difference was computed as [Performance (in-time) − Performance (out-of-time)]/[Performance (in-time) + Performance (out-of-time)], for each subject separately and then averaged.

We applied double-pulse TMS at 4 different time windows (Fig. 1*C*): Before target onset (T1; −53 to −13 ms), around target onset (T2; −13 to +27 ms), shortly after target onset (T3; +27 to +67 ms), and slightly later after target onset (T4; +67 to +107 ms). We hypothesized that, compared with the no-TMS condition, the detection advantage of in-time targets should diminish in at least one of the time windows if TMS over V5 affects the prediction effect.

**Figure 3.**
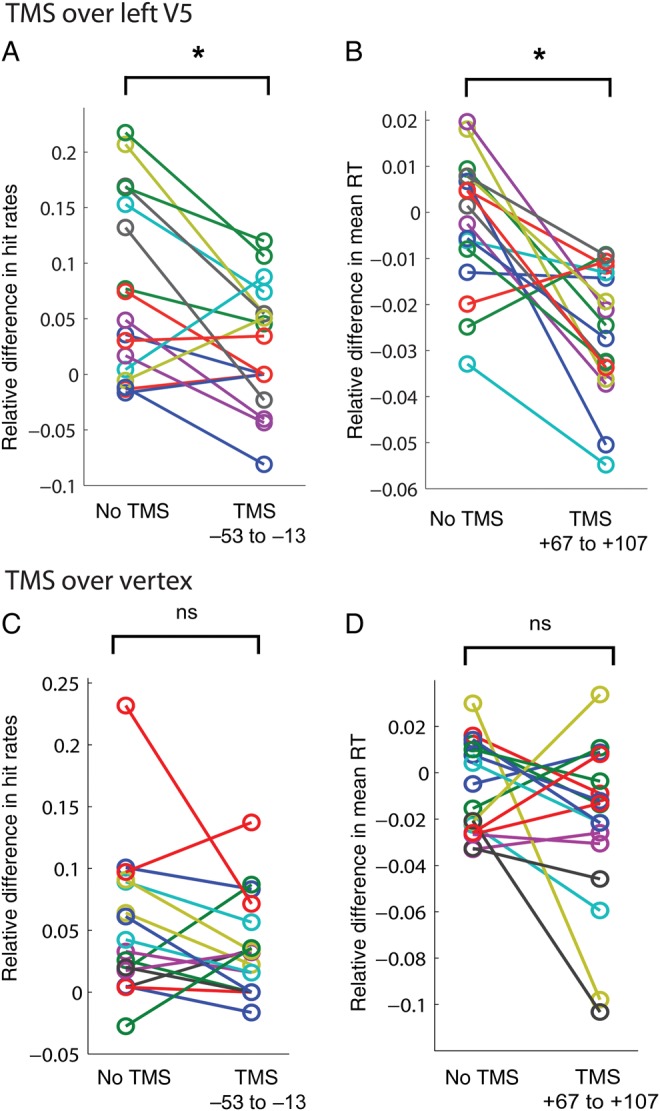
Individual subject data for the critical time windows. (*A*) Individual subject data for the significant TMS effect during V5 stimulation, that is, relative hit rate difference in the no-TMS condition and T1. (*B*) Individual subject data for relative RT difference in the no-TMS condition and T4 during V5 stimulation. (*C*) Individual subject data for the comparison between no-TMS condition and T1, equivalent to *A*, for vertex stimulation. (*D*) Individual subject data for relative RT difference for the no-TMS condition compared with V4, equivalent to *B*, for TMS over the vertex.

Relative differences in hit rates were positive (i.e., in-time targets were detected better than out-of-time targets) in the no-TMS condition and in time windows T2, T3, and T4 (1-sample *t*-tests against zero, *P* < 0.003, Bonferroni-corrected *α*-level: 0.01), except for time window T1 (Fig. 2*A*). In T1, hit rates were not significantly different between the 2 target conditions (*P* > 0.05). Relative hit rate differences were significantly diminished in T1 compared with the no-TMS condition (paired-sample *t*-tests, *P* = 0.008, Bonferroni-corrected *α*-level: 0.0125), while not differing in all other time windows (*P* > 0.05). Data from individual subjects for this comparison are plotted in Figure 3*A*. Note that during vertex stimulation (Fig. 3*C*), relative hit rates did not differ significantly between the no-TMS condition and T1 (*P* > 0.05).

Relative differences in reaction times did not significantly depart from zero in the no-TMS condition, T1, T2, and T3 (*P* > 0.05, Bonferroni-corrected), but reaction times were significantly faster for in-time than out-of-time targets in T4 (*P* < 0.001), resulting in a negative difference (Fig. 2*C*). There was a tendency in the same direction in T2 (*P* = 0.03, uncorrected). Relative RT differences were significantly larger in T4 compared with the no-TMS condition (*P* < 0.001 Bonferroni-corrected *α*-level: 0.0125). This effect is plotted in Figure 3*B* for individual subjects, note that there was no such effect during vertex stimulation (Fig. 3*D*). There was a tendency for larger RT differences in T3 when compared with the no-TMS condition (*P* = 0.058, uncorrected).

In summary, double-pulse TMS over left V5 at 13–53 ms before target onset eliminated the detection advantage of in-time targets and significantly diminished the detectability difference between in-time and out-of-time targets compared with when no TMS was applied. In addition, TMS increased mean reaction time differences when applied 67–107 ms after target onset compared with the no-TMS condition.

**Figure 4.**
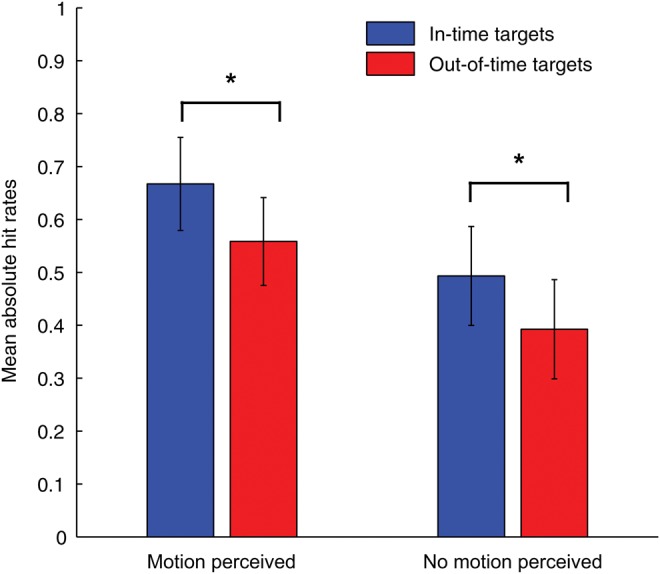
Results of the behavioral control experiment. Mean absolute hit rates for predictable in-time and out-of-time targets for trials when subjects reported to have consciously perceived apparent motion and when they report no apparent motion perception (just flicker) (*n* = 15). Error bars indicate SEM, stars indicate *P* < 0.05.

We performed a behavioral control experiment to determine to what extent the in-time/out-of-time effect depended on conscious apparent motion perception. To this aim, we broke the continuous apparent motion display down to separate trials and asked subjects to report both on target presence and on apparent motion perception. Subjects reported seeing apparent motion on average in 55.4% of trials (SEM 0.085). Figure 4 depicts mean absolute hit rates for each type of targets both when apparent motion was perceived and when it was not perceived. Note that overall mean hit rates were slightly lower in the control experiment than in the TMS experiment due to the fact that targets occurred in a lower proportion of trials, and that no TMS pulse indicated the potential presence of a target. Replicating the results of the TMS experiment and previous studies (Schwiedrzik et al. 2007; Vetter et al. 2012), predictable in-time targets were better detected than unpredictable out-of-time targets (repeated-measures ANOVA, *F*_1,14_ = 12.0, *P* = 0.004). This effect was independent from whether apparent motion was perceived or not (planned paired-sample *t*-tests: motion perceived: *P* = 0.004; no motion perceived: *P* = 0.027). Hit rates did not differ significantly between motion and no motion trials (*F*_1,14_ = 3.2, *P* > 0.05), and there was no interaction between target type and motion perception.

This experiment showed that our predictability effect is independent from conscious apparent motion perception and occurs even when subjects report not having perceived apparent motion.

## Discussion

In this study, we investigated whether hMT/V5 contributes to cortical predictions in the context of apparent motion. We stimulated left V5 with double-pulse TMS before, during, and after the presentation of predictable or unpredictable targets on the apparent motion trace. We found that the detection advantage of predictable targets, reliably present without TMS, is eliminated when TMS is applied 53–13 ms before target onset. That is, interfering with V5 processing shortly before target onset diminishes a prediction effect that has previously been demonstrated to be effective in V1 (Alink et al. 2010). While we did not measure feedback directly, the timing of our effect and previous results suggest that it is the feedback signal from V5 to V1 that is disrupted by TMS, occurring before target onset so to affect target processing. Thus, our results are direct evidence that the prediction of a future event can be affected by TMS.

Both Sterzer et al. (2006) and Wibral et al. (2009) provided evidence that the creation of an illusory motion token on the apparent motion trace, and thus a spatio-temporal prediction along the path, is likely to be caused by feedback processing from V5 to V1. Sterzer et al. (2006) demonstrated with effective connectivity analysis that increased BOLD activity levels in V1 at the retinotopic location of the apparent motion path were related to enhanced feedback connectivity between V5 and V1. Wibral et al. (2009) showed with electroencephalography that, during apparent motion, early visual areas are reactivated about 20–60 ms after the apparent motion-induced V5 component is detectable. However, both studies only provided correlational evidence for V5 feedback to V1 in an apparent motion setting. Due to our experimental manipulation of target presentation, our TMS results provide first causal evidence for apparent motion-induced V5 activity being necessary for a specific prediction effect, more precisely the interference with a prediction-related motion-unmasking effect (see below). Given that this prediction effect has been shown to modulate V1 activity (Alink et al. 2010), it is likely that TMS disrupted the predictive signaling from V5 to V1.

The timing of our effect fits well to other studies investigating V5 feedback onto V1. Wibral et al. (2009) demonstrated that apparent motion-induced feedback from V5 to early visual cortices occurs between 20 and 60 ms after V5 activity. In line with this, our results show that a disruption of V5 processing has an effect on predictable target detection within the same time frame (between 13 and 53 ms after V5 perturbation). Furthermore, Pascual-Leone and Walsh (2001) showed that moving phosphene perception induced by V5 TMS is reduced by a subthreshold V1 pulse at 5–45 ms after V5 stimulation. Similarly, Silvanto et al. (2005) demonstrated that V5 TMS 10–50 ms before a V1 TMS pulse, which normally induces a stationary phosphene, produced a moving phosphene. For real motion perception, single-pulse TMS over V5 has a disruptive effect at 30–40 ms before stimulus onset (Sack et al. 2006). Taken together with our results, it seems that the time frame of 5–60 ms after V5 activity is critical for feedback processing from V5 to V1.

While these previous studies provide evidence for the role of these connections for visual awareness, in line with electrophysiological studies in monkeys (e.g., Bullier 2006), our results further stress the role of V5 in forming a predictive model of potential sensory inputs. As such they also corroborate other studies showing that V5 is not only implicated in real motion perception (Sack et al. 2006; Cowey et al. 2006; Laycock et al. 2007) and the perception of moving phosphenes (Pascual-Leone and Walsh 2001; Silvanto et al. 2005), but also in motion priming (Campana et al. 2006) and illusory motion perception (Stewart et al. 1999; Matsuyoshi et al. 2007), 2 phenomena that are linked to predictive coding.

It should be noted that in our study, rather than interfering with the detection of an actually moving stimulus as done by many of the above-cited studies, we interfered with the detection of a stationary target that was presented within the context of illusory motion. In line with the predictive coding framework, we propose that the context of flashing stimuli creates an internal model in V5 that predicts when and where an illusory motion token can be expected on the apparent motion trace. Stimulating V5 before target onset briefly disrupts this internal model (or, at least, makes it imprecise) and interferes with the spatio-temporal-specific predictive signal, eliminating the detection advantage of predictable in-time targets over unpredictable out-of-time targets.

Another way of looking at our effect is the following. Apparent motion is known to induce masking on the apparent motion trace, that is, salient visual stimuli presented on the apparent motion traces are less readily detectable than outside of the trace (e.g., Yantis and Nakama 1998; Schwiedrzik et al. 2007; Hidaka et al. 2011). Despite this masking effect, in-time targets are better detected than out-of-time targets because they fit the predicted motion dynamics better and thus, counteract the masking effect (Schwiedrzik et al. 2007). Briefly, disrupting the internal predictive model by V5 TMS removes the motion-masking and makes in-time and out-of-time targets equally easy to be detected.

TMS can have both disruptive and facilitatory effects on detection, depending on the time point of stimulation relative to the perceptual or cognitive process at hand (Silvanto and Muggleton 2008) and depending on both the stimulus strength and the TMS strength (Schwarzkopf et al. 2011). In our case of the early time window, we propose that TMS has a disruptive effect due to the following reasoning. Given that we presented a stationary rather than a moving target, target detection per se should have happened in V1 and not in V5. Contrary to previous studies (Sack et al. 2006; Cowey et al. 2006; Laycock et al. 2007; Schwarzkopf et al. 2011), TMS over V5 should, therefore, not have interfered with actual target detection, but with the context in which the target appears, that is, with the internal motion model created in V5. If TMS had a facilitatory effect on the internal motion model, the difference between in-time and out-of-time detection rates should be greater than in the other time windows or than during vertex stimulation. The data show clearly that this was not the case.

Matsuyoshi et al. (2007) found that the perception of apparent motion at a frequency of 6–8.5 Hz is compromised after V5 activity was suppressed by offline low-frequency rTMS. Thus, it is conceivable that the elimination of our prediction effect could have been caused by the elimination of apparent motion perception through V5 stimulation. Given the experimental design of the TMS experiment (ongoing apparent motion stimulation, no indication of the start and end of a trial), it was not practicable to ask subjects after each trial whether they perceived apparent motion.

We therefore conducted an additional behavioral experiment to determine to what extent our predictability effect depends on conscious apparent motion perception. The results demonstrate that in-time targets are detected better than out-of-time targets, regardless of whether subjects perceived apparent motion or not. Therefore, whether TMS interfered with conscious apparent motion perception or not is not of concern to our main finding, as the predictability effect seems to operate independently of apparent motion perception. This finding is interesting by itself: it shows that the dynamics of apparent motion induce an internal predictive model about when and where an illusory motion token could be expected, independent from whether that illusory motion token is consciously perceived. This hints to the idea that predictive processes can be independent of consciousness (Den Ouden et al. 2009; Hohwy 2012).

Apart from an effect in detection rates at the early time window, we also found an effect of V5 TMS in reaction times in the late time window of 67–107 ms after target onset. Instead of an elimination of the prediction effect, however, a facilitation effect was found. Predictable targets were detected not only more frequently than unpredictable targets, but also reliably faster with less variance (see error bars [SEM] in Fig. 2*C*). Note that without TMS, no such difference in reaction times was observed (although it was present across all conditions as a main effect). It seems that TMS in this later time window either boosted the detection speed of in-time targets or slowed down the detection of out-of-time targets, while not affecting relative hit rates. Absolute reaction times do not allow us to decide between the 2 alternatives (absolute reaction times for both out-of-time and in-time targets did not differ significantly between TMS conditions). We think that it is more plausible to argue for a disruptive TMS effect, slowing down reaction times for out-of-time targets due to the following reasoning.

First, in the late time window, TMS was applied during or after the perceptual process of detecting targets in an apparent motion context, rather than before, and thus more likely to be disruptive (Silvanto and Muggleton 2008). Secondly, TMS is likely to introduce neural noise to the system (Schwarzkopf et al. 2011). Out-of-time targets, as opposed to in-time targets, cause an error signal as they do not match the predicted time and location of the illusory motion token, and are thus treated as noise by the internal predictive model. Additional noise through TMS is, therefore, more likely to slow down out-of-time target detection than boost in-time target detection. We propose that we see this effect most reliably in the latest time window (and only as a trend in the earlier time windows), because target detection by V1 and feed-forward signaling to V5 takes some time and may only be reliably completed after 67 ms (Foxe and Simpson 2002).

In summary, our results support the idea that predictive coding depends on feedback signals from higher cortical areas (Rao and Ballard 1999; Friston 2010). Within a well-controlled paradigm, our data show that visual prediction effects known to occur in V1 are significantly influenced by higher visual areas, such as V5. V5 may not be the only higher cortical area playing a role in apparent motion-induced prediction signals in V1; also V3a or parietal regions might be implicated in such feedback processing (Goebel et al. 1998; Sterzer et al. 2002; Claeys et al. 2003; Maus et al. 2010). Further research is required to elucidate the network of feedback connections implicated in predictive coding and motion-masking effects. Nevertheless, our results are one of the few direct demonstrations for predictive signals being created in a higher cortical area, and as such our results provide experimental support for predictive coding in the visual system.

## Supplementary Material

Supplementary material can be found at: http://www.cercor.oxfordjournals.org/.

## Funding

This work was funded by BBSRC grant BB/G005044/1 and by an ERC grant StG 2012_311751—BrainReadFBPredCode (Brain reading of contextual feedback and predictions). Funding to pay the Open Access publication charges for this article was provided by the European Research Council (ERC grant StG 2012_311751-BrainReadFBPredCode).

## Supplementary Material

Supplementary Data
