# Supplementary material for: TMS Over V5 Disrupts Motion Prediction
Source: Cereb Cortex. 2013 Oct 23;25(4):1052–9. doi: 10.1093/cercor/bht297 (PMC4380002; doi:10.1093/cercor/bht297)
Supplement: Supplementary Data [file supp_25_4_1052__index.html]

TMS Over V5 Disrupts Motion Prediction — TMS Over V5 Disrupts Motion Prediction — Supplementary Data 

# TMS Over V5 Disrupts Motion Prediction

## Supplementary Data

Supplementary Data

**Files in this Data Supplement:**

- Supplementary Table 1 - docx file
- Supplementary Video 1 - avi file
- Supplementary Video 2 - avi file
