# Supplementary material for: TMS Over V5 Disrupts Motion Prediction
Source: Cereb Cortex. 2013 Oct 23;25(4):1052–9. doi: 10.1093/cercor/bht297 (PMC4380002; doi:10.1093/cercor/bht297)
Supplement: Supplementary Data [file supp_bht297_bht297supp_table1.docx]

**Supplementary Table 1.** Absolute mean hit rates and reaction times for the TMS experiment.

|  | **No TMS** | **TMS**  **– 53 to -13** | **TMS**  **-13 to +27** | **TMS**  **+27 to +67** | **TMS**  **+67 to +107** |
| --- | --- | --- | --- | --- | --- |
| Mean hit rate [%]  (SEM)  **In-time** | 67.65  (3.62) | 82.43  (2.99) | 83.37  (3.10) | 86.17  (2.62) | 84.39  (3.07) |
| Mean hit rate [%]  (SEM)  **Out-of-time** | 59.20  (4.33) | 78.01  (2.55) | 72.80  (3.22) | 75.88  (3.16) | 76.35  (3.40) |
| Mean RT [ms]  (SEM)  **In-time** | 500.81  (12.11) | 430.92  (23.51) | 426.04  (22.59) | 434.21  (20.05) | 417.20  (21.36) |
| Mean RT [ms]  (SEM)  **Out-of-time** | 503.03  (12.83) | 440.55  (20.64) | 444.16  (20.98) | 449.05  (23.19) | 439.20  (22.32) |
